# Cognitive function in patients with myelin oligodendrocyte glycoprotein antibody-associated disease

**DOI:** 10.1007/s00415-025-13582-3

**Published:** 2026-01-06

**Authors:** Rebekka Rust, Susanna Asseyer, Patrick Schindler, Claudia Chien, Sophia Rekers, Carsten Finke, Frederike Cosima Oertel, Klemens Ruprecht, Sven Jarius, Brigitte Wildemann, Velina Chavarro, Tanja Schmitz-Hübsch, Friedemann Paul, Pia Sophie Sperber

**Affiliations:** 1 Experimental and Clinical Research Center (ECRC), Charité – Universitätsmedizin Berlin, corporate member of Freie Universität Berlin, Humboldt-Universität zu Berlin, Berlin, Germany; 2 Neuroscience Clinical Research Center (NCRC), Charité – Universitätsmedizin Berlin, corporate member of Freie Universität Berlin, Humboldt-Universität zu Berlin, Berlin, Germany; 3 Institute for Immunology, Charité – Universitätsmedizin Berlin Campus Virchow-Klinikum (CVK), Augustenburger Platz 1, 13353 Berlin, Germany; 4 German Center for Cardiovascular Disease (DZHK), partner site, Berlin, Germany; 5 Department of Neurology, Charité – Universitätsmedizin Berlin, corporate member of Freie Universität Berlin and Humboldt-Universität zu Berlin, Berlin, Germany; 6 Department of Psychiatry and Neurosciences, Charité – Universitätsmedizin Berlin, corporate member of Freie Universität Berlin and Humboldt-Universität zu Berlin, Berlin, Germany; 7 Berlin School of Mind and Brain, Humboldt-Universität zu Berlin, Berlin, Germany; 8 Division of Neuroimmunology, Department of Neurology, University of Heidelberg, Heidelberg, Germany; 9 Department of Neurosurgery, Ochsner Health System, New Orleans, LA USA

**Keywords:** Cognitive function, Myelin oligodendrocyte glycoprotein antibody-associated disease (MOGAD), Symbol digit modalities test (SDMT), Brief repeatable battery of neuropsychological tests (BRB-N)

## Abstract

**Background:**

Data on cognition in adult patients with myelin oligodendrocyte glycoprotein antibody-associated disease (pwMOGAD) are scarce.

**Objective:**

To examine cognitive function in pwMOGAD and assess relative risks (RR) for cognitive impairment (CImp) in pwMOGAD relative to healthy controls (HC), aquaporin 4-immunoglobulin G positive neuromyelitis optica spectrum disorders (pwAQP4+NMOSD), and double-seronegative NMOSD (pwdsNMOSD) compared to HC.

**Methods:**

Data derived from a cohort with neuroimmunological disorders. Cognitive performance was assessed using Rao’s brief repeatable battery of neuropsychological tests, compared to HC using confounder-adjusted linear regressions. CImp was defined as performing two standard deviations below the HC mean in any subtest. RR for CImp was calculated using generalized linear models.

**Results:**

We evaluated cognitive performance of 21 pwMOGAD and 25 HC. CImp was additionally determined in 43 pwAQP4+NMOSD and 15 pwdsNMOSD. PwMOGAD performed worse on Selective Reminding Test, and the symbol digit modalities test compared to HC. Adjusted RR for CImp were 1.9 (95% CI 0.9–4.1) in pwMOGAD, 1.9 (95% CI 1.0–3.9) in pwAQP4+NMOSD and 2.1 (95% CI 0.9–4.6) in pwdsNMOSD.

**Conclusion:**

pwMOGAD performed worse in information processing speed, verbal learning, storage and retrieval compared to HC. RR for CImp in pwMOGAD compared to HC was similar to that estimated for pwAQP4+NMOSD and pwdsNMOSD.

**Supplementary Information:**

The online version contains supplementary material available at 10.1007/s00415-025-13582-3.

## Introduction

Myelin oligodendrocyte glycoprotein antibody-associated disease (MOGAD) is an inflammatory demyelinating disease of the CNS with monophasic or relapsing course [1, 2]. Clinical manifestations of MOGAD comprise optic neuritis, transverse myelitis, brainstem or cerebellar encephalitis, cortical encephalitis, and acute disseminated encephalomyelitis with substantial overlap with neuromyelitis optica spectrum disorders (NMOSD) despite different pathophysiology [3, 4]. Recently, international diagnostic criteria for MOGAD have been proposed based on distinctive radiologic, histopathological, laboratory and clinical features [1, 5].

Several studies have demonstrated cognitive impairment in NMOSD, primarily affecting attention, information processing speed (IPS), memory, and verbal fluency with prevalence rates between 19 and 67%, partly due to varying definitions of cognitive impairment [6, 7]. However, data on cognitive performance in MOGAD remain sparse. One study of 17 MOGAD and 20 aquaporin 4 immunoglobulin G-positive (AQP4+) NMOSD patients found both groups showed lower performance in verbal learning, IPS, and cognitive screening (Montreal Cognitive Assessment) compared to healthy controls (HC) [8], with hippocampal atrophy associated with clinical disability and cognitive impairment in MOGAD but not in AQP4+NMOSD patients [8]. Reduced verbal reasoning and slower overall response time were also noted in individuals with pediatric-onset relapsing MOGAD [9].

In a just recently published study, MOGAD patients showed deficits in semantic fluency and congruent speed compared to normative data from HC [10]. 11% (11/99) of these patients showed impairment in two or more neuropsychological tests. Disease manifestation with cerebral lesions seems to be an important factor for reduced visual IPS and semantic fluency, according to that study [10].

In the present study, we aimed to (i) determine the extent and characteristics of possible cognitive deficits in MOGAD patients compared to HC using an internationally established neuropsychological test battery and (ii) compare the risk of cognitive impairment in MOGAD to that in AQP4+NMOSD, double-seronegative (ds) NMOSD and HC.

## Methods

### Cohort description and ethics

Patients with MOGAD were participants of a prospective single-center observational cohort of patients with NMOSD and related diseases, including MOGAD (Berlin NMO Cohort Study), conducted at the Charité—Universitätsmedizin Berlin, Neuroscience Clinical Research Center (NCRC). Data for these analyses, however, were derived from one time-point only as cognitive data during follow-up were limited, rendering this analysis cross-sectional. Inclusion period of this study was from 05/16/2013 until 08/18/2021. Inclusion and exclusion criteria for this cohort study are listed in the supplemental material. Study visits were conducted outside of an acute attack, i.e., at least three months from symptom onset. All participants gave written informed consent prior to participation in the NMO Cohort Study. The cohort was approved by the local ethics committee (EA1/041/14) and was conducted in line with guidelines framed by the declaration of Helsinki.

### Inclusion and exclusion criteria for the present analysis

Inclusion criteria for the present patient selection from the NMO Cohort Study were for MOGAD patients: (i) age of at least 18 years, (ii) diagnosis of MOGAD with a typical clinical syndrome, presence of serum myelin oligodendrocyte glycoprotein (MOG)-immunoglobulin G (IgG) and the exclusion of red flags and alternative diagnoses as defined by criteria by the international recommendations [1]. Additionally, MOGAD patients needed to fulfill also the MOGAD consensus criteria proposed by Banwell et al. [5]. From this cohort, we also included patients with AQP4+NMOSD or dsNMOSD. All of these patients met the International Panel for NMO Diagnosis (IPND) 2015 criteria [11]. HC were recruited via a local announcement on the Charité—Universitätsmedizin Berlin intranet to establish local reference values for a cognitive test battery. Eligibility required age ≥ 18 years and all HC were assessed by neurologists from the author team, including a detailed medical history and brief examination, ensuring no past or current neurological or psychiatric conditions. All patients and HC gave written informed consent. The participation was approved by the local ethics committee. For our present analysis, we considered only NMO Cohort Study visits conducted before 01/03/2020, since the protocol of the cohort for cognitive testing changed after this date. The cognitive data from the symbol digit modalities test (SDMT) and Paced Auditory Serial Addition Test (PASAT) of the MOGAD patients used here have already been included in a recently published analysis [10].

### Clinical characterization and study assessments

We assessed demographics (age, sex), educational status (in three categories, referring to the German school system [lower Secondary (9 years of school attendance), Secondary (10 years), High school (12–13 years)]), immunotherapy, clinical disability [using the Expanded Disability Status Scale (EDSS) performed by trained physicians] [12], and number of attacks since first manifestation of the disease. Depressive symptoms were assessed with the German Version of the revised Beck Depression Inventory (BDI) I and BDI II and fatigue with the Fatigue Severity Scale (FSS) [13, 14]. The BDI II scores range from 0 (best) to 63 (worst) (0–8: no depressive symptoms; 9–13: minimal mood disturbance; 14–20: mild depressive symptoms, 21–28: moderate depressive symptoms; ≥ 29: severe depressive symptoms). A relevant depressive syndrome was defined by a BDI I score ≥ 13 or a BDI II score ≥ 14 [13, 15]. We categorized the FSS into non‐fatigue (FSS ≤ 4.0), moderate fatigue (> 4.0 & < 5.0) and severe fatigue (FSS ≥ 5.0) [16]. Participants underwent MRI, clinical, and cognitive assessments on the same day as Rao’s brief repeatable battery of neuropsychological tests (BRB-N) testing whenever possible, or as close to that date as feasible.

### Antibody measurement and serostatus determination

Serum samples were obtained during regular study visits and immediately stored at − 80° until testing. MOG- and AQP4-IgG were measured with indirect immunofluorescence at EUROIMMUN (Lübeck, Germany) using fixed cell-based assays (CBA) overexpressing the respective antigen, as previously described [17]. MOG-IgG results and double-seronegative serostatus were confirmed at the Molecular Neuroimmunology Group at the University of Heidelberg using an in-house fixed CBA (cut-off 1:10; detection method: Fc-specific anti-human IgG secondary antibody) and/or at University Medicine Innsbruck using an in-house live CBA (cut-off 1:160; detection method: H + L-specific anti-human IgG secondary antibody), both utilizing transfected HEK293 cells expressing recombinant human full-length MOG as antigenic substrates and non-transfected HEK293 cells as control substrate [18, 19]; only samples positive in at least two assays were deemed positive for the purpose of this study [17, 20]. Patients were considered MOG-IgG- or AQP4-IgG-positive if results were positive at any visit or based on documented results from previous tests.

### Cognitive assessment

Trained staff assessed a shortened German version of the BRB-N for all patients [21]. Visits for the HC were conducted from November 2012 to December 2013, using the same cognitive assessment protocol [22]. The protocol included the Selective Reminding Test (SRT) with Long-Term Storage (SRT-LTS) and Consistent Long-Term Retrieval (SRT-CLTR), measuring verbal learning, storage and retrieval; the Spatial Recall Test (SPART), measuring visual spatial learning, storage and retrieval; the three-seconds Paced Auditory Serial Addition Test (PASAT-3) [23], measuring IPS, working memory, divided attention (by an auditory stimulus) and sustained attention. After a delay interval of approximately 30 min, the delayed recall of the SRT (SRT-DR) and the SPART (SPART-DR) were assessed. This was followed by the symbol digit modalities test (SDMT) [24], measuring IPS, working memory, and sustained attention (by a visual stimulus); and the word list generation (WLG) measuring verbal association fluency. The tests were conducted in the order listed here to optimize the timing for assessments with delayed recall. Only the baseline BRB-N assessment was used to avoid learning bias. We defined impaired cognitive function (CF) as scores below two standard deviations (SD) compared to the HC mean in any test. The rationale for this definition were two previous NMO studies [25, 26].

### Magnet resonance imaging and assessment of visual acuity

The MRI protocol was described previously [27]. Brain lesion segmentation on T2-weighted fluid-attenuated inversion recovery images was performed manually by experienced radiology technicians using ITK-SNAP (http://www.itksnap.org/pmwiki/pmwiki.php). Lesion counts and volumes were extracted using FSL software (https://fsl.fmrib.ox.ac.uk/fsl/docs/#/statistics/index). Deep gray matter (DGM) volumes (i.e., left and right thalamus, nucleus caudate, putamen, pallidum, hippocampus, amygdala, nucleus accumbens) and brainstem volume were obtained from lesion-filled magnetization-prepared rapid gradient echo scans using FSL FIRST in milliliter (ml) [28]. High-contrast visual acuity was acquired under best correction with Early Treatment in Diabetes Retinopathy Study charts at 20ft distance with retro-illuminated charts. This method was only applied after 2016, which renders, that best corrected visual acuity was only available in a subset of patients.

### Statistical analysis

Group characteristics were described by means and medians with corresponding measures of variance [SD and boundaries of the inter quartile range (IQR)]. Total DGM volume was calculated as the sum of all DGM sub-volumes (brainstem not included). We calculated absolute standardized mean differences, to evaluate how well the groups are balanced regarding baseline characteristics [29]. Normality of residuals from cognitive tests was assessed using kernel density estimation. We compared cognitive performance between MOGAD patients and HC using crude and age-, sex-, and education-adjusted linear regression models. Resulting β effect estimates were calculated with corresponding 95% confidence intervals (CI). Estimates were interpreted based on study population variation: effects ≥ 1 SD from the mean were large, 0.5 SD medium, and ≤ 0.25 SD small [30]. In a second analysis, we estimated the relative risk (RR) for cognitive impairment in MOGAD, AQP + NMOSD and dsNMOSD patients compared to HC using generalized linear models, with log link, robust error variance, and adapted Poisson family distribution for precise CI [31, 32]. In a sensitivity analysis, we excluded patients who showed impaired SDMT performance and significant visual impairment, defined as an EDSS visual sub-score greater than three.

We explored relationships of mean FSS, BDI values, total T2 lesion and total DGM volume, and from both eyes averaged logarithm of the Minimum Angle of Resolution (logMAR) visual acuity with the SDMT in the total cohort and in MOGAD patients only (except for visual acuity because of sparse data). We used the BDI I for the total cohort and BDI II for the MOGAD patient subgroup because BDI I was available in only four MOGAD patients.

T2 lesion volumes (in ml) were log-transformed for analysis. For this purpose, a small constant of 0.001 was added to all values because some individuals had a total lesion volume of 0 ml. Data were visualized with scatter plots with regression lines, and correlation coefficients were calculated. Additionally, we calculated linear regression models with BDI and FSS, or depressive syndrome (yes/no), fatigue defined by cut-offs (see above), and total log-transformed T2 lesion volume as independent variables and the SDMT as dependent variable, respectively.

Data cleaning, preparation, and visualization were conducted with R version 4.2.1, generalized linear models were calculated in STATA 14, StataCorp LLC Texas USA.

### Data availability statement

Data and code scripts are available from the corresponding author upon reasonable request. Due to data protection regulations, the research data cannot be uploaded to a data repository. This study followed the STROBE Statement for Reporting.

## Results

### Description of the cohort

**Fig. 1:**
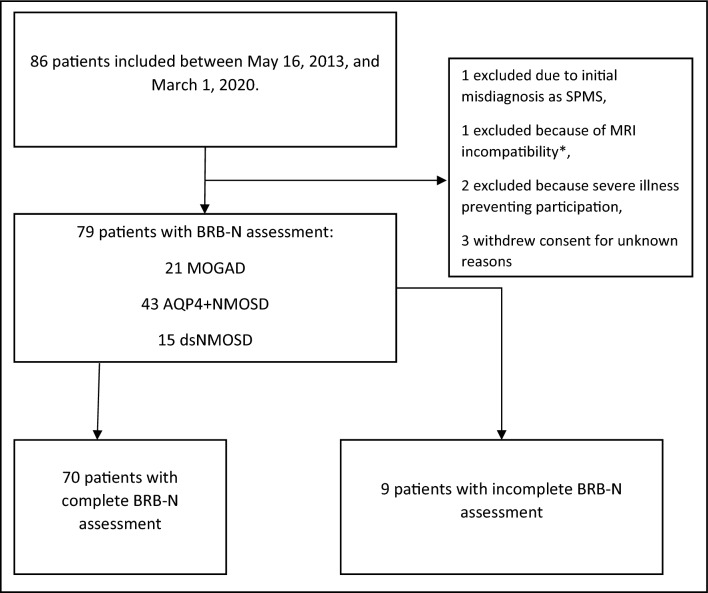
Study flowchart. *AQP4+*, aquaporin 4 immunoglobulin G positive; *BRB-N* brief repeatable battery of neuropsychological tests, *ds* double seronegative, *MRI* magnet resonance tomography imaging, *MOGAD* myelin oligodendrocyte glycoprotein antibody-associated disease, *NMOSD* neuromyelitis optica spectrum disorder, *SPMS* secondary progressive multiple sclerosis. *MRI incompatibility (e.g., metal implant, pacemaker, obesity) was exclusion criteria for the NMO Cohort Study

A total of 86 patients were enrolled between May 16, 2013 and March 01, 2020. After excluding seven patients who were excluded due to screening failures or consent withdrawal, 79 patients were eligible for analysis (see Fig. 1). Most of the assessments were conducted on the same day of BRB-N-Testing. Only in 12 individuals MRI scans were conducted around the BRB-N assessment on a mean + 14 (sd = 34) days after.

Among these, 21 had MOGAD, 43 had AQP4+NMOSD, and 15 had dsNMOSD. MOGAD patients had a mean age of 44 years (SD = 15), a median EDSS of 2.5 (IQR = 2–3) and a mean of eleven cerebral T2 lesions (SD = 9), with a median lesion volume of 1.1 ml (range 0.4–2.8 ml). Six MOGAD patients (28.6%) showed a relevant depressive syndrome based on BDI I or BDI II scores. The mean fatigue score in MOGAD patients was 3.65 (SD = 1.63). Compared to NMOSD patients, MOGAD patients experienced more attacks but had a lower EDSS score and less cerebral lesion load. The HC group comprised 25 individuals with a mean age of 48 years (SD = 14), and had a higher male proportion and a higher educational level than the patient groups. Eleven patients with neuro-immunological disorder (MOGAD: *n* = 2, AQP4+NMOSD: *n* = 8, dsNMOSD: *n* = 1) had a significant visual impairment defined by a raw visual function sub-score > 3 in the EDSS. Please see Table 1 for the main demographic and clinical characteristics of the study participants and Supplemental Fig. 1 for corresponding pairwise ASMD. Characteristics were quite imbalanced across groups, indicated by high ASMD values for almost all pairs.

**Table 1:** Baseline characteristics

	HC (*N* = 25)	MOGAD (*N* = 21)	AQP4+NMOSD (*N* = 43)	dsNMOSD (*N* = 15)	Overall (*N* = 104)
Sex					
Female *n* (%)	6 (24.0%)	14 (66.7%)	40 (93.0%)	10 (66.7%)	70 (67.3%)
Age					
Years, mean (SD)	41.5 (12.9)	43.5 (14.8)	48.2 (14.1)	45.8 (14.6)	45.3 (14.1)
School education					
Lower secondary *n* (%)	1 (4.0%)	2 (9.5%)	4 (9.3%)	1 (6.7%)	8 (7.7%)
Secondary *n* (%)	6 (24.0%)	8 (38.1%)	20 (46.5%)	7 (46.7%)	41 (39.4%)
High school *n* (%)	18 (72.0%)	11 (52.4%)	12 (27.9%)	4 (26.7%)	45 (43.3%)
Missing *n* (%)	0 (0%)	0 (0%)	7 (16.3%)	3 (20.0%)	10 (9.6%)
Time since onset					
Years, mean (SD)	–	8.03 (11.0)	5.98 (6.31)	4.57 (5.21)	6.27 (7.74)
Missing *n* (%)	–	0 (0%)	4 (9.3%)	0 (0%)	4 (9.6%)
EDSS					
Median [IQR]	–	2.25 [2, 3]	4 [2.5–5]	3.50 [2.50, 5]	3.50 [2–4]
Missing *n* (%)	–	1 (4.8%)	1 (2.3%)	1 (6.7%)	4 (5.0%)
Number of attacks since onset					
Mean (SD)	–	3.95 (2.68)	3.86 (3.02)	2.71 (1.54)	3.65 (2.69)
Missing, *n* (%)	–	2 (9.5%)	7 (16.3%)	1 (6.7%)	9 (11.3%)
Immuno-therapy					
None, *n* (%)	–	6 (28.6%)	3 (7.0%)	3 (20.0%)	12 (11.5%)
Azathioprine, *n* (%)	–	2 (9.5%)	12 (27.9%)	3 (20.0%)	17 (16.3%)
Methotrexate, *n* (%)	–	2 (9.5%)	1 (2.3%)	0 (0%)	3 (2.9%)
Rituximab, *n* (%)	–	7 (33.3%)	19 (44.2%)	6 (40.0%)	32 (30.8%)
Prednisolone, *n* (%)	–	3 (14.3%)	0 (0%)	1 (6.7%)	4 (3.8%)
MMF, *n* (%)	–	1 (4.8%)	2 (4.7%)	1 (6.7%)	4 (3.8%)
Glatiramer acetate, *n* (%)	–	0 (0%)	1 (2.3%)	1 (6.7%)	2 (1.9%)
Belimumab, *n* (%)	–	0 (0%)	1 (2.3%)	0 (0%)	1 (1.0%)
Missing, *n* (%)	–	0 (0%)	4 (9.3%)	0 (0%)	4 (5%)
BDI					
No depressive syndrome, *n* (%)	–	15 (71.4%)	24 (55.8%)	6 (40.0%)	45 (43.3%)
Depressive syndrome, *n* (%)	–	6 (28.6%)	12 (27.9%)	8 (53.3%)	26 (25.0%)
Missing, *n* (%)	–	0 (0%)	7 (16.3%)	1 (6.7%)	8 (10.0%)
FSS mean values					
Mean (SD)	–	3.65 (1.63)	3.89 (1.96)	4.41 (1.48)	3.93 (1.78)
Missing, *n* (%)	–	1 (4.8%)	4 (9.3%)	0 (0%)	5 (6.3%)
Fatigue					
No fatigue, *n* (%)		13 (61.9%)	18 (41.9%)	7 (46.7%)	38 (36.5%)
Fatigue, *n* (%)		2 (9.5%)	8 (18.6%)	2 (13.3%)	12 (11.5%)
Severe fatigue, *n* (%)		5 (23.8%)	13 (30.2%)	6 (40.0%)	24 (23.1%)
MRI T2 lesions					
Count mean (SD)	–	11.4 (22.0)	31.6 (35.7)	21.0 (22.4)	24.1 (31.2)
Volume ml median (IQR)	–	0.3 (0.1–1.0)	1.1 (0.4–2.8)	0.5 (0.1–2.8)	0.7 (0.2 –2.0)
Missing, *n* (%)	–	0 (0%)	1 (2.3%)	0 (0%)	1 (1.3%)
Total DGM					
Volume in ml mean (sd)	–	46.2 (5.41)	45.6 (3.72)	47.4 (3.99)	46.1 (4.27)
Missing, *n* (%)	–	1 (4.8%)	2 (4.7%)	0 (0%)	(26.9%)
Best corrected decimal visual acuity*					
Median (IQR)		1.30 (1.08–1.51)	1.06 (0.93–1.22)	0.90 (0.90–1.25)	1.13 (0.90–1.43)
Missing, *n* (%)	–	6 (28.6%)	37 (86.0%)	6 (40.0%)	49 (62.0%)

*AQP4+NMOSD* aquaporin 4 immunoglobulin G positive neuromyelitis optica spectrum disorder (NMOSD), *BDI* Beck Depression Inventory; relevant depressive syndrome according to BDI I ≥ 13 or BDI II ≥ 14/no depressive syndrome according to either BDI I < 13 or BDI II < 14, *DGM* deep gray matter, *dsNMOSD* double-seronegative NMOSD, *EDSS* Expanded Disability Status Scale, *FSS* Fatigue Severity Scale, *HC* healthy controls, *MOGAD* myelin oligodendrocyte glycoprotein antibody-associated disease, *Max* maximal value, *Min* minimal value, *MMF* Mycophenolate Mofetil, *MRI* magnet resonance tomography imaging, *n* number, *SD* standard deviation, *IQR* boundaries of the interquartile range. No missing data in age and sex. Fatigue: no fatigue (FSS ≤ 4.0), fatigue (4.0 < FSS < 5.0) and severe fatigue (FSS ≥ 5.0); total DGM, deep gray matter volume. Clinical characteristics were not assessed in HC. Proportion of missing is calculated from only those with assessments according to protocol (i.e., excluding HC in clinical characteristics [*n* = 79]). Number of attacks counted until BRB-N assessment

*High proportion of missing data due to protocol changes

### Cognitive performance

**Fig. 2:**
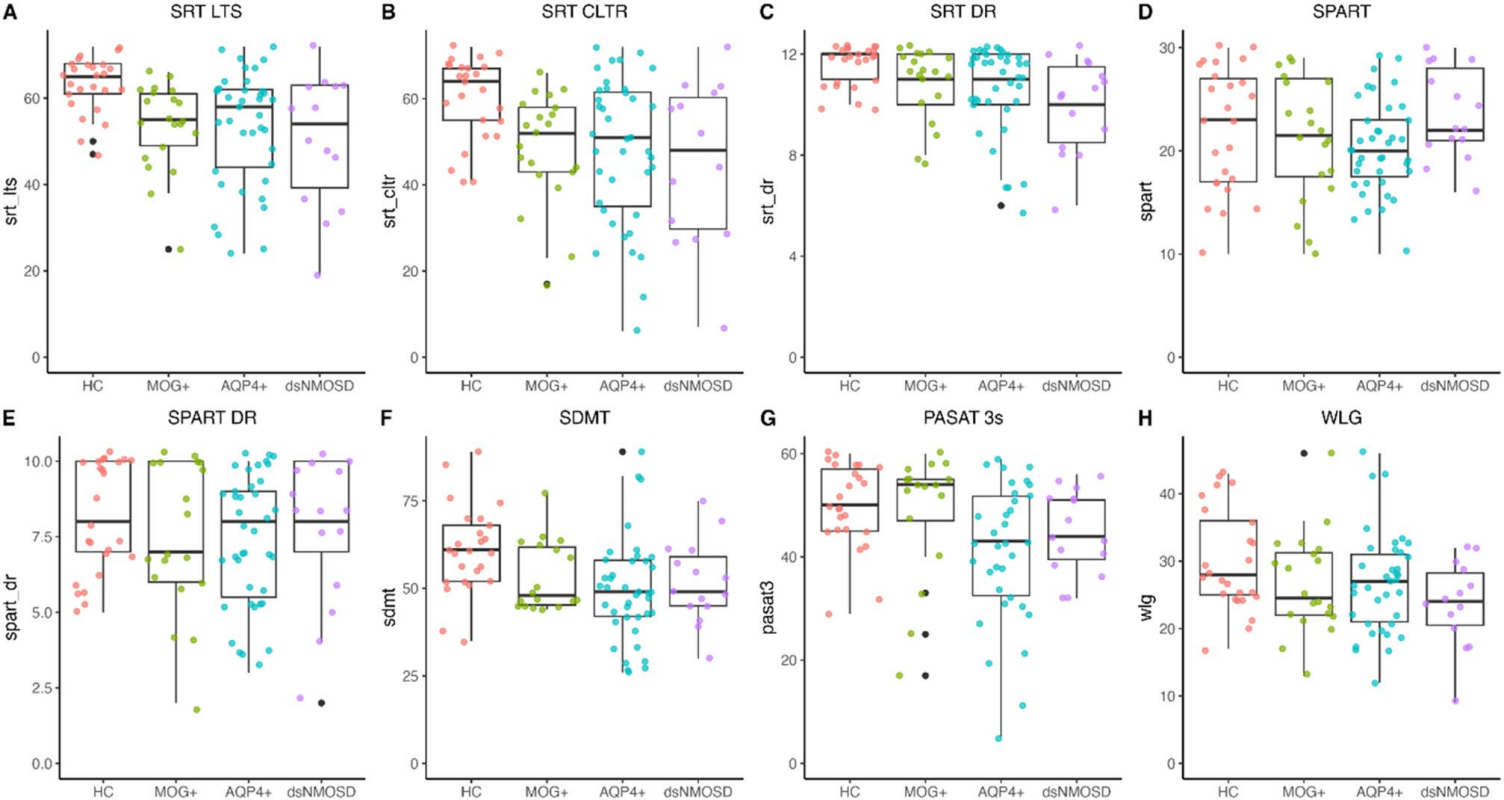
Visualization of raw values of each test from the brief repeatable battery of neuropsychological tests stratified according to different disease groups and healthy controls. *SRT-LTS sum* sum total of the Selective Reminding Test (SRT) long-term storage (LTS), *SRT-CLTR sum* sum total of SRT Consistent Long-Term Retrieval (CLTR), *SRT-DR* SRT delayed recall (DR), *SPART sum* sum total of the Spatial Recall Test (SPART), *SPART-DR* SPART delayed recall, *PASAT 3s* Paced Auditory Serial Addition Test—3 second version, *SDMT* symbol digit modalities test, *WLG* word list generation, *AQP4+* aquaporin 4 immunoglobulin G positive neuromyelitis optica spectrum disorder (NMOSD), *dsNMOSD* double-seronegative NMOSD, *HC* healthy controls, *MOG+* myelin oligodendrocyte glycoprotein antibody-associated disease

Figure 2 illustrates cognitive performance across all groups on the BRB-N measures. MOGAD patients performed worse than HC in the SRT-LTS (*β* = − 9.62 [95% CI − 14.96–4.28], SRT-CLTR (*β* = − 9.73 [95% CI − 16.76–2.70]), SRT-DR (*β* = − 0.82 [95% CI − 1.51–0.13]), and SDMT (*β* = − 7.23 [95% CI − 14.40–0.04]). Differences in PASAT, SPART, and WLG were not considered clinically important due to small effect sizes and wide CI. All results are summarized in Table 2.

**Table 2:** Results from linear regression models comparing each test result of the brief repeatable battery of neuropsychological tests of myelin oligodendrocyte glycoprotein antibody-associated disease patients to healthy controls

Test	Range (sd)	Unadjusted		Adjusted for age, sex, education	
		*β*	95% CI	*β*	95% CI
**SRT-LTS sum**	**25–72 (9.48)**	− **9.55**	**− 14.47 to − 4.62**	**− 9.62**	**− 14.96 to − 4.28**
**SRT-CLTR sum**	**17–72 (12.54)**	**− 11.55**	**− 18.25 to − 4.85**	**− 9.73**	**− 16.76 to − 2.70**
**SRT-DR**	**8–12 (1.11)**	**− 0.76**	**− 1.40 to − 0.13**	**− 0.82**	**− 1.51 to − 0.13**
SPART sum	10–30 (5.95)	− 0.92	− 4.55 to 2.71	0.45	− 3.06 to 3.96
SPART-DR	2–10 (2.14)	− 0.59	− 1.91 to 0.72	− 0.16	− 1.36 to 1.04
PASAT 3 s	17–60 (10.06)	− 1.31	− 7.77 to 5.15	0.55	− 6.16 to 7.25
**SDMT**	**35–89 (12.02)**	**− 7.80**	**− 14.99 to − 0.62**	**− 7.23**	**− 14.40 to − 0.04**
WLG	13–46 (7.68)	− 3.27	− 7.86 to 1.32	− 0.73	− 5.70 to 4.25

Range, minimum and maximum values from all comparators

*sd* standard deviation, *β* beta effect estimate comparing performance of patients with myelin oligodendrocyte glycoprotein antibody-associated disease (MOGAD) to healthy controls (HC), *95% CI* 95% confidence interval, *SRT-LTS sum* sum total of the Selective Reminding Test (SRT) long-term storage, *SRT-CLTR sum* sum total of SRT Consistent Long-Term Retrieval, *SRT-DR* SRT delayed recall, *SPART sum* sum total of the Spatial Recall Test (SPART), *PASAT 3 s* Paced Auditory Serial Addition Test—3 s version, *SDMT* symbol digit modalities test, *WLG* word list generation

Statistically significant results based on an alpha level of 0.05 are bold in this table

Overall, 10 (55%) MOGAD, 23 (66%) AQP4+NMOSD, 9 (60%) dsNMOSD patients, and 7 (28%) HC showed cognitive impairment. The adjusted RR for cognitive impairment was 1.9 (95% CI 0.9–4.1) for MOGAD, 1.9 (95% CI 1.0–3.9) for AQP4+NMOSD, and 2.1 (95% CI 0.9–4.6) for dsNMOSD (see Table 3 and Supplemental Table 1 for the conducted tests). Impairments in SRT tests were the most frequent across all disease groups. Among patients with significant visual impairment two AQP4+NMOSD patients showed also reduced performance on the SDMT (i.e., < 2 SD below the mean, according to our definition). We excluded these individuals in a sensitivity analysis, and the results remained essentially unchanged (data not shown).

**Table 3:** Risk ratios calculated from generalized linear models for the effect of the different neuro-immunological disease entities on impairment in cognitive tests

Group (*n*)	Cognitively impaired	Unadjusted		Model 1: adjusted for age, sex, education	
	*n* (%)	RR	95% CI	RR	95% CI
HC (25)	7 (28)	1	(ref.)	1	(ref.)
MOGAD (21)	11 (55)	1.98	0.93–4.22	1.94	0.93–4.05
AQP4+NMOSD (35)	23 (66)	2.35	1.19–4.62	1.93	0.95–3.90
dsNMOSD (15)	9 (60)	2.14	1.01–4.57	2.09	0.94–4.62

Cognitively impaired, defined as scoring lower than two standard deviations on at least one test of the brief repeatable battery of neuropsychological tests, *AQP4+* aquaporin 4 immunoglobulin G positive, *ds* double seronegative, *HC* Healthy controls, *MOGAD* myelin oligodendrocyte glycoprotein antibody-associated disease, *NMOSD* neuromyelitis optica spectrum disorder, *ref.* reference, *RR* risk ratios, *95% CI* 95% confidence intervals

### Association of fatigue, depression, cerebral lesion load, total deep gray matter volume and averaged visual acuity with information processing speed

**Fig. 3:**
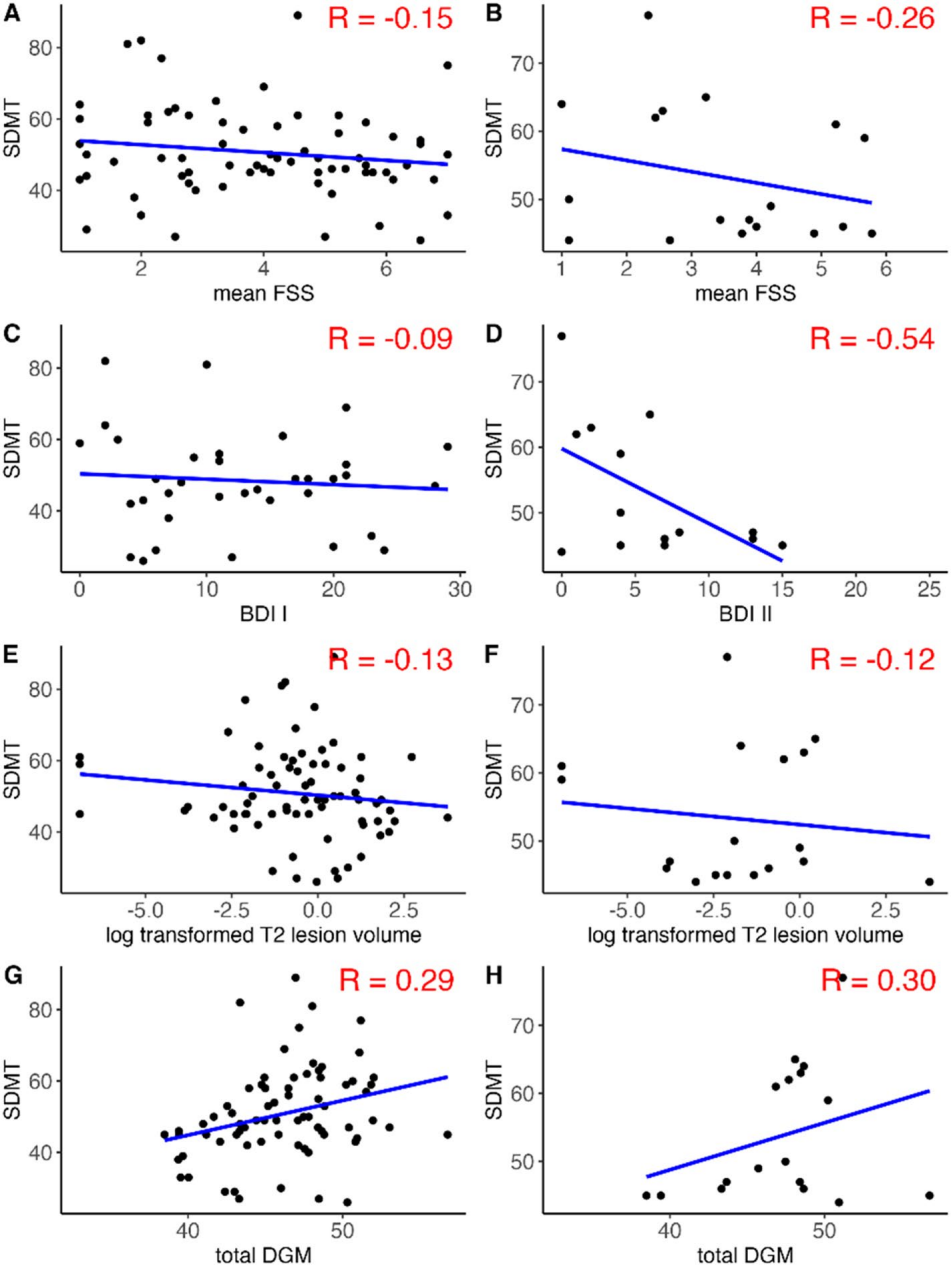
Scatter plots with linear regression lines and correlation coefficients between fatigue, depression, T2 lesion and deep gray matter volume and small digit reminding test values. *SDMT* symbol digit modalities test, *BDI I and II* Becks Depression Inventory I and II, *FSS* Fatigue Severity Scale, *HC* healthy controls, *MOGAD* myelin oligodendrocytic glycoprotein antibody-associated disease. *R* correlation coefficient, *log-transformed T2 lesion volume* logarithmic transformed cerebral T2 lesion volume (ml), *total DGM* total deep gray matter volume. Left column (**A**, **C**, **E**, **G**) for the total disease population, and right column (**B**, **D**, **F**, **H**) for MOGAD only. *n* number, for **A** *n* = 69, **B** *n* = 18, **C** *n* = 36, **D** *n* = 14, **E** *n* = 73, **F** *n* = 18, **G** *n* = 72, **H** *n* = 17

Associations and correlation coefficients of fatigue, depressive symptoms, T2-lesion and total DGM volume with SDMT are shown in Fig. 3 [left column (A, C, E, G) for the total disease population, and right column (B, D, F, H) for MOGAD only]. In patients with MOGAD and not in the total population, a negative correlation (*R* = − 0.54) was observed between BDI values and SDMT scores. Results from linear and log-linear models are summarized in Table 4. In summary, in patients with MOGAD, however not in the total disease population, a negative correlation (*R* = − 0.54) was observed between BDI values and SDMT scores. This relationship was not confirmed when using depressive syndrome as a binary predictor (*β* = − 1.93, 95% CI − 15.59–11.72). In the total disease population, total DGM volume was associated with SDMT, however, not in MOGAD alone. Visual acuity on a logMAR scale was not correlated with SDMT in 30 individuals with available data (data not shown). In this analysis, sample size was too small for group stratification.

**Table 4:** Associations of disease outcomes and characteristics with information processing speed in patients with neuro-immunological disease and MOGAD patients

	Neuro-immunological disease (MOGAD, AQP4+NMOSD, dsNMOSD)		MOGAD patients only	
	*β*	95% CI	*β*	95% CI
FSS	− 1.10	− 2.82 to 0.62	− 1.65	− 4.92 to 1.62
BDI (I or II)	− 0.15	− 0.77 to 0.47	− 1.15	− 2.26 to − 0.03
T2 lesion volume	− 0.86	− 2.35 to 0.63	− 0.47	− 2.51 to 1.56
Total DGM volume	0.97	0.20–1.73	0.69	− 0.50 to 1.88

*AQP4+* aquaporin 4 immunoglobulin G positive, *ds* double seronegative, *MOGAD* myelin oligodendrocyte glycoprotein antibody-associated disease, *NMOSD* neuromyelitis optica spectrum disorder, *BDI I and II* Becks Depression Inventory I and II, *95% CI* 95% confidence intervals, *FSS* Fatigue Severity Scale, *total DGM* total deep gray matter volume

## Discussion

This single-center cross-sectional study revealed that MOGAD patients performed worse in verbal learning, storage and retrieval (SRT tests), IPS, and attention (SDMT) compared to HC. Additionally, the risk for cognitive impairment across all BRB-N tests was double in MOGAD compared to HC, similar to results for AQP4+NMOSD and dsNMOSD.

Our results are in line with one study showing MOGAD-related lower performance in verbal learning and visual IPS [8] though the CogniMOG study did not report similar verbal learning deficits [10]. Interestingly, while our MOGAD cohort performed worse than HC on the SDMT, the differences fell short of our impairment threshold. Notably, our cognitive impairment rate (55%) under a one-test impairment definition was higher than that in the CogniMOG study (32%), likely due to our use of the comprehensive BRB-N and different impairment cut-offs [10].

The cut-off for cognitive impairment in our study was set at two standard deviations below the mean in at least one test, consistent with two previous NMOSD studies [23, 28]. Although the threshold is relatively low, nearly 30% of our HC also showed impairment in a single test, raising questions about the applicability of this definition in the general population. Defining impairment based on a single test is relatively liberal and may overestimate prevalence. While the absolute count warrants caution, between-group comparisons are unlikely to be affected, as the threshold was applied uniformly (i.e., misclassification was nondifferential). Currently, the BRB-N is one of the most broadly used comprehensive neuropsychological test battery in neuro-immunological diseases [21, 33, 34], and evaluates varied cognitive domains. This study is the first to apply the full BRB-N in MOGAD, offering a broader assessment than previous screening methods (e.g., MuSIC as a cognitive screening test for German-speaking countries), providing broader insight and enabling international comparability across cognitive domains [21]. However, lacking German normative data for BRB-N, we used a center-specific HC group. Therefore, a center-specific HC comparison group was obtained at our study center. Despite adjustments for demographic differences, demographic disparities between HC and MOGAD groups (sex ratio, education) remain a limitation and may bias the results. Additionally, differences were observed in nearly all characteristics, as indicated by the ASMDs. However, it is elusive whether these differences reflect disease manifestations or are due to small group sizes.

Mild visual deficits, undetectable via EDSS, could influence test results with a visual component [35], although our data do not support such an influence, as visual acuity was not associated with SDMT, as previously reported [26]. For populations with visual deficits, a test excluding visual stimuli might better assess IPS.

Structural brain alterations, including loss of thalamic and hippocampal volume in MOGAD patients [36], gray matter atrophy in frontal and temporal lobe, insula, thalamus, and hippocampus, and white matter fiber disruption in optic radiation and anterior/posterior corona radiate [8], could contribute to cognitive deficits in MOGAD in different domains in CF. Although our MOGAD patients showed lower cerebral MRI lesion load than AQP4+NMOSD and dsNMOSD, lesion load alone does not consistently predict cognitive impairment in NMOSD [37], while only limited data is available for MOGAD. In fact, DGM volume was associated with SDMT performance in the overall disease cohort, but not in MOGAD, possibly due to the small sample size. The differences in total DGM volume between disease groups were minor compared to other characteristics. Functional and longitudinal MRI studies could add clarifying the impact of functional or structural brain changes on cognitive function in MOGAD in future.

Due to insufficient longitudinal data, analyses were restricted to a cross-sectional design; fluctuations in cognitive performance were therefore not considered. Evidence from recent studies suggests that cognitive deficits in MOGAD are generally mild and non-progressive, with transient impairments observed in some, only pediatric-onset patients [38], but no systematic longitudinal change demonstrated over 2 years indicating that cognitive deficits in MOGAD are stable over time rather than progressive [10].

Fatigue and depression, common in MOGAD [39, 40], can affect cognitive performance. Consistent with previous studies, we found no relationship between fatigue (FSS) and cognition (SDMT) across the cohort or within MOGAD [10]. However, it contrasts previous results regarding NMOSD patients, which reported an association between fatigue (measured by the FSMC) and the SDMT [7]. In contrast, a moderate correlation was noted between BDI scores and SDMT in MOGAD patients, though BDI’s screening intent limits the reliability of this linear analysis. Overall, further investigation into how fatigue and depression intersect with cognitive deficits is warranted.

Cognitive disorders, especially in memory, attention, and IPS, have been reported in 19–67% of NMOSD patients, although data remain sparse [6, 7], and are less explored in MOGAD. Our findings suggest similar cognitive impairment rates and profiles in MOGAD, highlighting the need for further studies examining specific cognitive domains and tracking cognitive trajectories over time to aid in disease characterization and potential stratification.

## Strengths and limitations

A key strength of this study is the use of comprehensive BRB-N testing for cognitive evaluation in MOGAD, rather than reliance on screenings alone. The small number of MOGAD cases (*n* = 21) is due to the rarity of the disease and limits our analyses to exploratory comparisons for orientation rather than definitive conclusions. Main limitations include the lack of normative population data for the BRB-N and the demographic differences (sex ratio, education) between HC and MOGAD groups. Further limitations include a small sample size, and the absence of validation for BRB-N in MOGAD. Additionally, our results apply only to patients without major visual impairment, and longitudinal data from a more homogeneous patient group are needed to clarify cognitive impairment progression over disease stages as we have been tested at different stages of the disease, i.e., with largely varying disease durations.

## Conclusion

MOGAD patients demonstrated lower performance in verbal learning, memory and IPS (BRB-N SRTs and SDMT) than HC, with a cognitive impairment risk similar to AQP4+NMOSD and dsNMOSD.

## Supplementary Information

Below is the link to the electronic supplementary material.

Supplementary file1 (DOCX 240 KB)
