# Supplementary material for: Cognitive function in patients with myelin oligodendrocyte glycoprotein antibody-associated disease
Source: J Neurol. 2026 Jan 6;273(1):61. doi: 10.1007/s00415-025-13582-3 (PMC12775078; doi:10.1007/s00415-025-13582-3)
Supplement: Supplementary file 1 — Supplementary file1 (DOCX 240 KB) [file 415_2025_13582_MOESM1_ESM.docx]

**SUPPLEMENTAL MATERIAL**

**Cognitive Function in Patients with Myelin Oligodendrocyte Glycoprotein Antibody -**

**Associated Disease**

Rebekka Rust 1,2,3, Susanna Asseyer 1,2,5 Patrick Schindler 1,2,5, Claudia Chien 1,2, 6, Sophia Rekers 5,7, Carsten Finke 5, Frederike Cosima Oertel 1,2, Klemens Ruprecht 8, Sven Jarius 8, Brigitte Wildemann 8, Velina Chavarro 2,9, Tanja Schmitz-Hübsch 1,2, Friedemann Paul 1,2, Pia Sophie Sperber 1,2,4,5

1 Experimental and Clinical Research Center (ECRC), Charité – Universitätsmedizin Berlin, corporate member of Freie Universität Berlin, Humboldt-Universität zu Berlin, Germany

2 Neuroscience Clinical Research Center (NCRC), Charité – Universitätsmedizin Berlin, corporate member of Freie Universität Berlin, Humboldt-Universität zu Berlin, Germany

3 Institute for Immunology, Charité – Universitätsmedizin Berlin Campus Virchow-Klinikum (CVK), Augustenburger Platz 1; 13353 Berlin

4 German Center for Cardiovascular Disease (DZHK), partner site Berlin, Germany

5 Department of Neurology, Charité – Universitätsmedizin Berlin, corporate member of Freie Universität Berlin and Humboldt-Universität zu Berlin, Germany

6 Department of Psychiatry and Neurosciences, Charité – Universitätsmedizin Berlin, corporate member of Freie Universität Berlin and Humboldt-Universität zu Berlin, Germany

7 Berlin School of Mind and Brain, Humboldt-Universität zu Berlin, Germany

8 Division of Neuroimmunology, Department of Neurology, University of Heidelberg, Heidelberg, Germany

9 Ochsner Health System, Department of Neurosurgery, New Orleans Louisiana USA

Inclusion and exclusion criteria for the “NMO cohort”:

Inclusion criteria:

- Age between 18 and 70 years
- Presence of a disease from the neuromyelitis optica (NMO) spectrum (see below)
- Inclusion diagnoses from the NMO spectrum are:

- NMO according to Wingerchuk 2006

- aquaporin-4 immunoglobulin G positive (AQP4+) NMO according to Wingerchuk 2015

- aquaporin-4 immunoglobulin G negative (AQP4-) NMO according to Wingerchuk 2015

- longitudinal extensive transverse myelitis (LETM), recurrent optic neuritis

- Myelin oligodendrocyte glycoprotein IgG positive (MOG+)

Exclusion criteria:

- patients with a diagnosis of multiple sclerosis (MS) according to the McDonald 2010 criteria,
- a contraindication or other condition affecting retinal (optical coherence tomography) and
- contraindication or other condition affecting magnet resonance tomography imaging (MRI).
- clinically relevant concomitant diseases that would prevent participation in the study in the opinion of the investigator? (e.g. arterial hypertension not controlled by medication, severe diabetes mellitus, alcohol or drug abuse, chronic infectious diseases such as HIV or hepatitis)
- limitation to give informed consent, adhere to the rules of the protocol and complete the study
- pregnancy

Supplemental Table 1

Cognitive impairment of MOGAD, NMOSD patients and healthy controls in different cognitive tests

| Test abbr. | Healthy controls | MOGAD | AQP4+  NMOSD | AQP4-  NMOSD |
| --- | --- | --- | --- | --- |
|  | N (%) cognitive impaired | N (%) cognitive impaired | N (%) cognitive impaired | N (%) cognitive impaired |
| SRT LTS SUM | 2 (8) | 6 (29) | 12 (30) | 7 (50) |
| SRT CLTR SUM | 0 (0) | 4 (19) | 11 (28) | 5 (36) |
| SRT DR | 3 (12) | 6 (30) | 14 (35) | 8 (53) |
| SPART SUM | 1 (4) | 1 (5) | 1 (3) | 0 (0) |
| SPART DR | 0 (0) | 3 (16) | 5 (13) | 2 (13) |
| PASAT 3s | 2 (8) | 3 (18) | 9 (26) | 2 (13) |
| SDMT | 1 (4) | 0 (0) | 7 (18) | 1 (7) |
| WLG | 0 | 1 (5) | 1 (3) | 1 (7) |

AQP4+ NMOSD, aquaporin-4 immunoglobulin G Antibody seropositive neuromyelitis optica spectrum disorder; dsNMOSD, double seronegative NMOSD; MOGAD, Myelin Oligodendrocytic Glycoprotein antibody associated disease; HC, healthy controls; PASAT 3s, Paced Auditory Serial Addition Test – 3 second version; SDMT, Symbol Digit Modalities Test; SPART SUM, sum total of the Spatial Recall Test (SPART); SPART DR, SPART delayed recall; SRT LTS SUM, sum total of the selective reminding test (SRT) long term storage; SRT CLTR SUM, sum total of SRT Consistent Long Term Retrieval; SRT DR, SRT delayed recall; WLG, word list generation; SD, standard deviation.

Supplemental Figure 1

Pairwise absolute standardized mean differences (ASMD) of characteristics of patients with neuroimmunological disorders and healthy controls


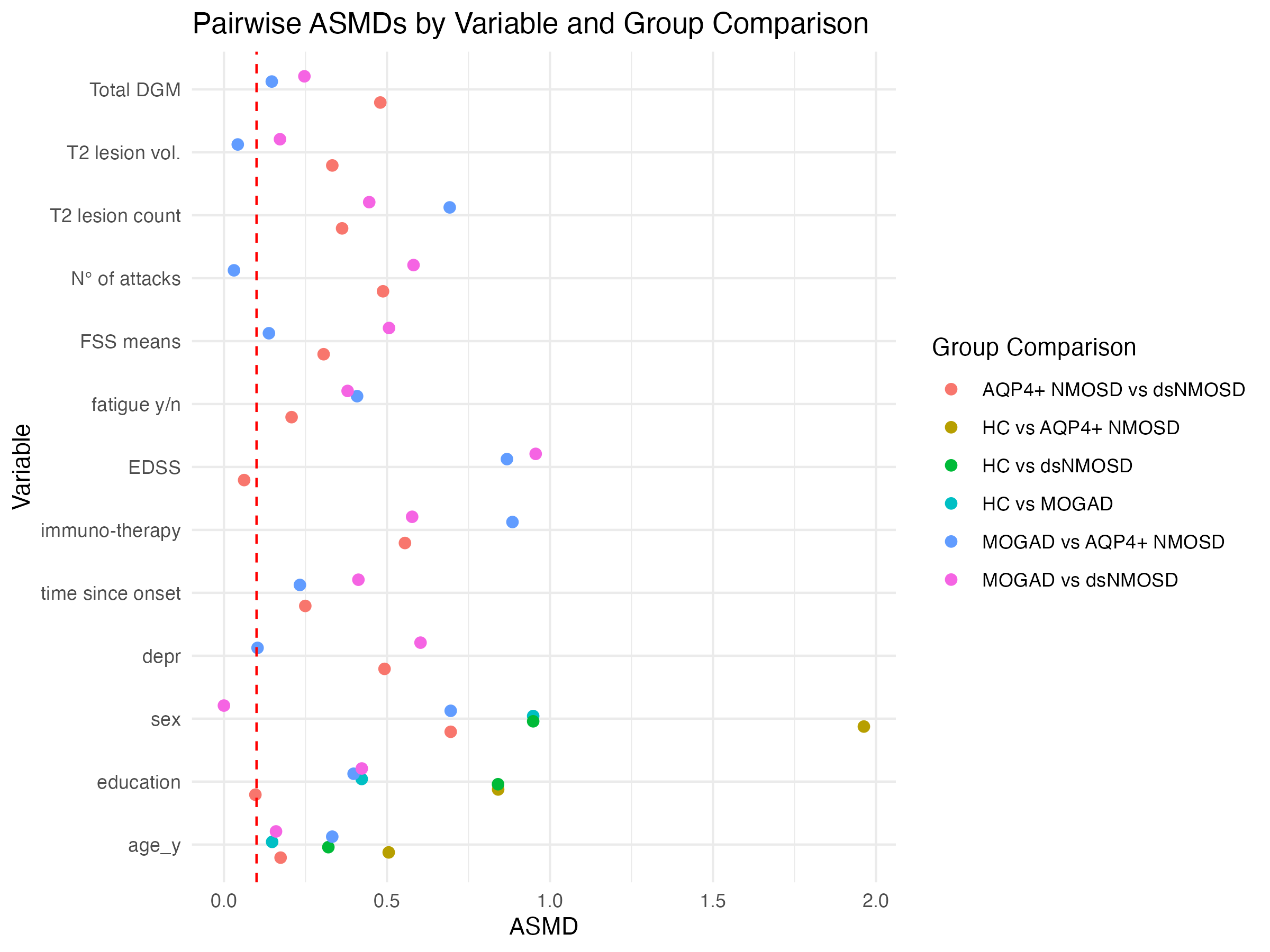


ASMD, absolute standardized mean differences; AQP4+ NMOSD, aquaporin-4 immunoglobulin G Antibody seropositive neuromyelitis optica spectrum disorder; dsNMOSD, double seronegative NMOSD; MOGAD, Myelin Oligodendrocytic Glycoprotein antibody associated disease; HC, healthy controls; DGM, volume of deep grey matter; FSS, fatigue severity scale; fatigue y/n: yes fatigue (FSS > 4.0), no fatigue (FSS ≤4.0); depr, depression yes/no; relevant depressive syndrome according to BDI-I ≥ 13 or BDI-II ≥ 14 / no depressive syndrome according to either BDI-I < 13 or BDI-II < 14; age and time since onset in years; N° of attacks, number of attacks since onset; vol., volume. Differences of the left side of the red dashed line are considered negatable. Visual acuity was not added due to high proportion of missing data.

Supplemental Table 2

Deep grey matter (DGM) volumes from cerebral magnet resonance images of individuals with neuroimmunological disorders

|  | **MOGAD (N=21)** | **AQP4+ NMOSD (N=43)** | **seron. NMOSD (N=15)** | **Overall (N=79)** |
| --- | --- | --- | --- | --- |
| **Left thalamus** mean (sd) | 7.84 (1.07) | 7.63 (0.65) | 7.84 (0.67) | 7.73 (0.78) |
| **Right thalamus** mean (sd) | 7.61 (1.03) | 7.43 (0.65) | 7.60 (0.61) | 7.51 (0.75) |
| **Thalamus total** mean (sd) | 15.5 (2.07) | 15.1 (1.27) | 15.4 (1.25) | 15.2 (1.51) |
| **Left caudate nucleus** mean (sd) | 3.36 (0.46) | 3.19 (0.39) | 3.54 (0.56) | 3.30 (0.46) |
| **Right caudate nucleus** mean (sd) | 3.61 (0.50) | 3.40 (0.40) | 3.65 (0.57) | 3.50 (0.47) |
| **Caudate nucleus total** mean (sd) | 6.97 (0.96) | 6.59 (0.76) | 7.19 (1.13) | 6.81 (0.92) |
| **Left putamen** mean (sd) | 4.72 (0.70) | 4.61 (0.59) | 4.85 (0.70) | 4.69 (0.63) |
| **Right putamen** mean (sd) | 4.72 (0.66) | 4.66 (0.56) | 4.89 (0.67) | 4.72 (0.60) |
| **Putamen total** mean (sd) | 9.45 (1.35) | 9.27 (1.11) | 9.74 (1.31) | 9.41 (1.21) |
| **Left pallidum** mean (sd) | 1.66 (0.26) | 1.70 (0.20) | 1.78 (0.17) | 1.71 (0.21) |
| **Right pallidum** mean (sd) | 1.73 (0.27) | 1.70 (0.18) | 1.77 (0.21) | 1.72 (0.21) |
| **Pallidum total** mean (sd) | 3.40 (0.51) | 3.40 (0.36) | 3.55 (0.37) | 3.43 (0.41) |
| **Left hippocampus** mean (sd) | 3.81 (0.50) | 3.74 (0.44) | 3.85 (0.26) | 3.78 (0.43) |
| **Right hippocampus** mean (sd) | 3.71 (0.75) | 3.80 (0.39) | 3.87 (0.35) | 3.79 (0.50) |
| **Hippocampus total** mean (sd) | 7.52 (1.10) | 7.54 (0.72) | 7.73 (0.56) | 7.57 (0.81) |
| **Left amygdala** mean (sd) | 1.25 (0.25) | 1.43 (0.31) | 1.44 (0.22) | 1.38 (0.29) |
| **Right amygdala** mean (sd) | 1.28 (0.21) | 1.45 (0.28) | 1.40 (0.20) | 1.39 (0.26) |
| **Amygdala total** mean (sd) | 2.53 (0.40) | 2.87 (0.56) | 2.84 (0.39) | 2.78 (0.51) |
| **Left nucleus accumbens** mean (sd) | 0.48 (0.11) | 0.46 (0.09) | 0.49 (0.10) | 0.47 (0.10) |
| **Right nucleus accumbens** mean (sd) | 0.41 (0.12) | 0.37 (0.11) | 0.38 (0.08) | 0.38 (0.10) |
| **Nucleus accumbens total** mean (sd) | 0.89 (0.20) | 0.83 (0.18) | 0.87 (0.16) | 0.85 (0.18) |
| **DGM total** mean (sd) | 67.7 (8.50) | 67.6 (5.50) | 69.4 (5.89) | 68.0 (6.43) |
| **Brainstem** mean (sd) | 21.5 (3.30) | 22.0 (2.42) | 22.1 (2.36) | 21.9 (2.64) |

AQP4+NMOSD, aquaporin-4 immunoglobulin G positive neuromyelitis optica spectrum disorder (NMOSD); seron. NMOSD, double seronegative NMOSD; MOGAD, myelin oligodendrocyte glycoprotein antibody-associated disease; n, number; SD, standard deviation; DGM total, total deep grey matter volume; Missing data were n=1(4.8%) in MOGAD and n=2(4.7%) in AQP4+NMOSD for all DGM volumes.
